# The protective effects of *Clerodendranthus spicatus* (Thunb.) C. Y. Wu extract on oxidative stress induced by 2,2'-azo (2-methylpropamidine) dihydrochloride in HL-1 mouse cardiomyocytes

**DOI:** 10.3389/fcvm.2022.984813

**Published:** 2022-09-09

**Authors:** Ying Li, Jia Wang, Jiahui Jiang, Xiang Li, Ming Wang

**Affiliations:** Department of Cardiology, Chongqing Hospital of Traditional Chinese Medicine, Chongqing, China

**Keywords:** HL-1 mouse, oxidative stress, cardiomyocytes, heart, AAPH

## Abstract

To investigate the protective effects of *Clerodendranthus spicatus* (Thunb.) C. Y. Wu extract (CSTE) on oxidative stress injury in HL-1 mouse cardiomyocytes induced by 2,2'-azo (2-methylpropamidine) dihydrochloride (AAPH, 1 mmol/L), HL-1 cells were co-cultured with different concentrations (10–100 μg/mL) of the CSTE for 24 h. A cell damage model was established by continuously culturing the cells in Dulbecco's Modified Eagle Medium plus AAPH for 4 h. Cell survival rates were measured by the 3-(4,5-dimethylthiazol-2-yl)-2,5-diphenyl-2H-tetrazolium bromide assay, and by measuring intracellular malondialdehyde (MDA) content. MDA and total reactive oxygen species (ROS) levels were determined by thiobarbituric acid colorimetry and the 2',7'-dihydrodichlorofluorescent sodium yellow diacetate probe, respectively. Apoptosis was measured by flow cytometry. The intracellular catalase (CAT), superoxide dismutase (SOD), glutathione peroxidase (GSH-Px), glutathione s-transferase (GST), γ-glutamylcysteine synthetase (γ-GCS), and glutathione (GSH) contents were determined by colorimetric methods. CSTE content was determined by high performance liquid chromatography. The CSTE pretreatment improved survival rates in damaged HL-1 cells, reduced total intracellular ROS and MDA levels, and reduced apoptosis. The CSTE also increased the activities of the antioxidant enzymes (CAT, SOD, GSH-Px, and GST), as well as the γ-GCS and GSH levels in damaged cells. Real-time fluorescence quantitative polymerase chain reaction analysis indicated that the CSTE upregulated CAT, SOD1, and GSH-Px mRNA expression levels. Additionally, the CSTE reduced MDA and ROS levels in HL-1 cells by improving the endogenous antioxidant system; thus, alleviating the oxidative stress damage caused by AAPH. Our compositional analyses revealed that the CSTE contained caffeic acid, isoquercetin, rosmarinic acid, luteolin, and baicalin. The CSTE demonstrates antioxidant and protective effects in myocardial cells.

## Introduction

**Figure 1:**
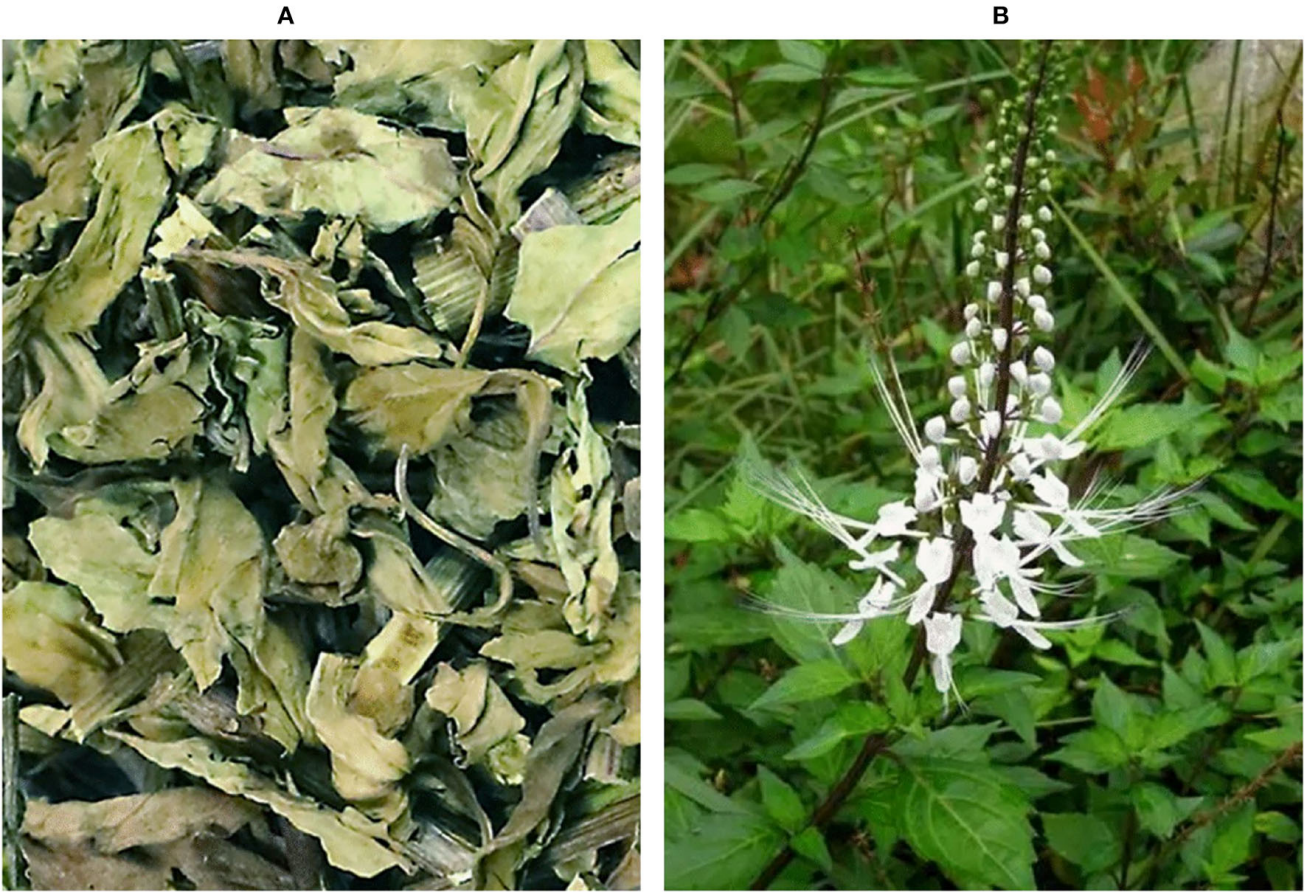
Observation diagram of *Clerodendranthus spicatus* (Thunb.) C. Y. Wu, **(A)** Dried *Clerodendranthus spicatus* (Thunb.) C. Y. Wu, commodity condition; **(B)** *Clerodendranthus spicatus* (Thunb.) C. Y. Wu in vegetative state.

*Clerodendranthus spicatus* (Thunb.) C. Y. Wu is a plant found in Asia and Oceania, it is a perennial herb in the *Lamiaceae* family (1), the aerial part of *Clerodendranthus spicatus* (Thunb.) C. Y. Wu is used as medicine or health tea (Figure 1). Previous studies have reported that a *C. spicatus* (Thunb.) C. Y. Wu extract (CSTE) exerts protective effects on some internal organs, including the kidneys, and protects against nephritis edema, hypertension, cholelithiasis hepatitis, rheumatoid arthritis, and inflammation of the fallopian tubes (2–4), which results in painful and poor urination, kidney atrophy, swollen hands and feet, and acute hepatitis; thus, the CSTE has been used as a traditional Chinese medicine in China (5). In addition, the CSTE may also have antibacterial and anti-inflammatory qualities when used in tea.

Superoxide anions (O${}_{2}^{-}$), peroxide ions (O${}_{2}^{2-}$), hydroxyl radicals (•OH), hydrogen peroxide (H_2_O_2_), peroxynitrite [ONOO^−^reactive oxygen species (ROS)], organic peroxyradicals (ROO•), and lipid peroxyradicals (LOO•) are physiological by-products of metabolism. Excessive accumulation of ROS in the body induces oxidative stress and causes disease. Endogenous antioxidant dysfunction in the heart leads to a high degree of oxidative stress in some chronic heart diseases (6, 7). Therefore, excessive ROS accumulation not only induces lipid peroxidation of polyunsaturated fatty acids in cell membranes but also degenerates macromolecules, such as proteins and genetic material in cells, leading to oxidative stress injury in cardiac cells and necrosis (8–10). Therefore, ROS are a significant pathogenic factor leading to chronic heart disease. Some plants used in traditional Chinese medicine have good antioxidant capacities, as they are rich in antioxidant molecules, and are often used to treat chronic diseases or play a preventive role as drinks. Therefore, ingesting this medicine (foods homologous to traditional Chinese medicine) helps improve antioxidant capacity, alleviates the adverse effects of oxidative stress on tissues and vital organs, and prevents the occurrence of related diseases (11). In some specific circumstances, ROS may be protective against ischemia-reperfusion injury during myocardial ischemia-reperfusion injury (12). In this study, only the common ROS-induced oxidative stress myocardial injury was investigated. The complex mechanism of ROS on cardiomyocytes needs further study.

HL-1 is a mouse cardiomyocyte cell line used to study oxidative stress-induced cardiomyocyte injury (13). We evaluated the potential preventative and health effects of the CSTE in combatting oxidative stress injury. Cardiomyocytes were treated with 2,2'-amidine hydrochloride (2,2'-azobis (2-methylpropionamidine) dihydrochloride (AAPH) to construct an oxidative damage cell model. We explored the protective mechanisms of the CSTE against oxidative stress induced by AAPH, and provide a theoretical reference point for the CSTE in preventing heart disease caused by oxidative stress.

## Materials and methods

### Extraction of *Clerodendranthus spicatus* (Thunb.) C. Y. Wu

*C. spicatus* (Thunb.) C. Y. Wu is produced in Sipsongpanna, Yunnan Province, China. The *C. spicatus* (Thunb.) C. Y. Wu from Sipsongpanna is the only one approved for medicinal use in China, so this study selects samples of this product. After the *C. spicatus* (Thunb.) C. Y. Wu (Kunming Xuanqing Biotechnology Co., Ltd, Kunming, Yunnan, China) was freeze-dried, it was crushed, ground, and sieved, and a particular amount of the sample powder was accurately weighed into a beaker. Ethanol (70%) was added at a liquid material ratio of 20:1. The solution was placed in a 60°C water bath for 2 h twice, suction filtrated, and the liquid was passed through FL-3 macroporous resin. The water and ethanol were removed from the resinous liquid using a rotary evaporator (evaporated until no liquid flowed into the beaker) and dried at a constant temperature of 60°C for 48 h. The dried sample was ground, weighed, sealed in an EP tube, and stored at 4°C.

### Cell culture and groups

HL-1 cardiomyocytes (National Collection of Authenticated Cell Cultures, Shanghai, China) were cultured in Dulbecco's Modified Eagle Medium (DMEM; Solarbio Life Science, Beijing, China) plus 10% fetal bovine serum and 1% cyanin-streptomycin dual antibody solution at 37°C in 5% CO_2_, and the medium was changed every 2 days. The cells (2 × 10^5^) were added to 96- and 6-well cell culture plates and cultured for the experiments. The oxidative damage cell model was prepared by continuously culturing the cells in DMEM plus 1 mmol/L AAPH for 4 h. Damaged cells (1 × 10^4^) were enumerated and added to wells containing 100 μL of the CSTE (10, 50, and 100 μg/mL) in 96-well plates for 24 h. Normal HL-1 cells without AAPH were used as the control group.

### 3-(4,5-Dimethylthiazol-2-Yl)-2,5-Diphenyl-2H-tetrazolium bromide (MTT) cell viability assay

Fifty 4-week-old female BALB/c mice (weight 20–25 g) were purchased from Hunan Slike. The HL-1 cells were treated and cultured for 24 h. Then, the medium was discarded, and 100 μL of MTT solution (0.5 mg/mL, Solarbio Life Science) was added to the cells for 4 h. The supernatant was discarded, 100 μL of dimethyl sulfoxide was added to the wells, the plate was shaken for 30 min, and the OD_490__nm_ value was measured. The cell survival rate was calculated using the formula: cell survival rate (%) = OD treatment group/OD normal group × 100.

### Cell malondialdehyde (MDA) production

After the treatments, MDA production was determined by thiobarbituric acid colorimetry. The cells were rinsed in phosphate-buffered saline (PBS), scraped, and lysed in precooled lysis solution. Then, 500 μL of the lysis supernatant, 15% trichloroacetic acid, and 0.67% thiobarbituric acid (400 μL, Solarbio Life Science) were added to a 5 mL tube. After mixing and incubating for 20 min at 95°C, the tubes were cooled, and 3 mL of isopropyl alcohol was added to extract the pigments. The OD_532nm_ value was measured, and total protein content in the cells was determined with a kit. MDA production was calculated according to the formula: MDA production (ng/mg Pro) = MDA content (ng/mL) × 1.5 mL/total protein weight (mg).

### Determination of intracellular ROS levels

After the cells were treated and grown in 6-well cell plates, Dulbecco's modified Eagle's medium (DMEM) plus 20 μmol/L 2,7-Dichlorodihydrofluorescein diacetate (DCFH-DA, Solarbio Life Science) was added for 20 min at 37°C. The cells were washed twice in cold PBS and absorbance was determined at an excitation wavelength of 485 nm. When the emission wavelength was 530 nm, the FLUOstar OPTIMA was used to measure fluorescence intensity, and relative ROS levels were calculated according to the formula: relative ROS content (%) = fluorescence intensity of the treatment group/fluorescence intensity of the normal group × 100.

### Determination of antioxidant enzyme activities, glutathione (GSH) levels, and γ-glutamylcysteine synthetase (γ-GCS) activity in cells

Cells (2 × 10^5^/well) were added to 6-well plates, treated, and cultured. An appropriate amount of SOD, catalase (CAT), GSH-Px, GSH, and γ-GCS (Shanghai Enzyme-linked Biotechnology Co., Ltd., Shanghai, China) were taken from the cell lysis solution. Enzyme activity was expressed in specific enzyme activity units (U/mg Pro), and GSH content and γ-GCS activity were expressed as μmol/mg Pro. The total protein content in the cells was corrected.

### Flow cytometry to detect cell death

After the cells were treated with MDLE, a 1 mL cell suspension (5 × 10^5^ cell/mL) was centrifuged at 4,500 rpm and 4°C, and the supernatant was discarded. The cells were rinsed in pre-chilled PBS and resuspended in 500 μL of PBS. The cells were mixed with 5 μL of Annexin V-FITC (ThermoFisher Scientific, Waltham, MA, USA) and 5 μL of propidium iodide and incubated in the dark at 37°C for 15 min (AccuriC6, BD Biosciences, San Jose, CA, USA). Flow cytometry was used to detect apoptosis (14).

### Antioxidant gene expression using real-time fluorescence quantitative polymerase chain reaction

Total RNA was extracted from the cells using Trizol reagent (ThermoFisher Scientific) and RNA concentrations were adjusted to the same levels after UV spectroscopy was performed to assess purity. RNA (2 μg) was added to the OligodT_18_, RNase, dNTPs, and MLV enzyme (1 μL each), and 5× Buffer (10 μL) in the PCR tubes. Then, cDNA was synthesized at 37°C for 120 min at 99°C for 4 min, and at 56°C for 3 min, and CAT, GSH-Px, and superoxide dismutase 1 (SOD1, Cu/Zn-SOD) mRNA expression levels were detected by RT-fluorescence qPCR. cDNA (2 μL, ThermoFisher Scientific), upstream and downstream primers (10 μmol/L), 10 μL of the SYBR Premix Ex Taq II (2×), and 0.4 μL of the ROX Reference Dye (50×) were added to the total reaction system (20 μL) and 5.6 μL of sterile double-distilled water was added and thoroughly mixed with the reagents and placed in the QuantStudio^TM^ 6 Flex PCR apparatus for the reaction. The amplification parameters were: 95°C for 35 s, 55–59°C for 30 s, 95°C for 15 s, 60°C for 60 s over 40 cycles, and 95°C for 15 s (SteponePlus, ThermoFisher Scientific). Amplifications were performed three times in parallel, and the mean cycle threshold (Ct) value was recorded. The expression level of the target gene (F) was calculated according to the following formula. The housekeeping gene glyceraldehyde-3-phosphate dehydrogenase (GAPDH) was used as an internal reference (15).


$$\begin{array}{l} F=\frac{2^{Ct_{1}-Ct_{2}}}{2^{Ct_{3}-Ct_{4}}} \end{array}$$

Where Ct_1−4_ are the Ct values of the genes in the samples, GAPDH in the test samples, genes in the blank samples, and GAPDH in the blank samples, respectively.

### High-performance liquid chromatography

The CSTE and standard samples were extracted with methanol, passed through 0.22 μm organic filter membranes, and transferred to brown liquid vials for testing. The composition of the CSTE was determined by HPLC (UltiMate3000, ThermoFisher Scientific) using an Accucore C18 column (5 μm, 4.6 × 250 mm). Mobile phase A was 0.5% acetic acid in water and mobile phase B was acetonitrile. The flow rate was 0.5 mL/min, and the column temperature was 30°C. The detection wavelength was 359 nm and the injection volume was 5 μL. Pre-equilibrium was performed for 10 min.

### Data analyses

All experiments were repeated three times, and the results are expressed as mean ± standard deviation. All statistical analyses were performed with SPSS 19.0 software (SPSS Inc., Chicago, IL, USA), and one-way analysis of variance was used to detect differences. A *P*-value < 0.05 was considered significant.

## Results

### Effects of the CSTE on the survival of HL-1 cells

**Figure 2:**
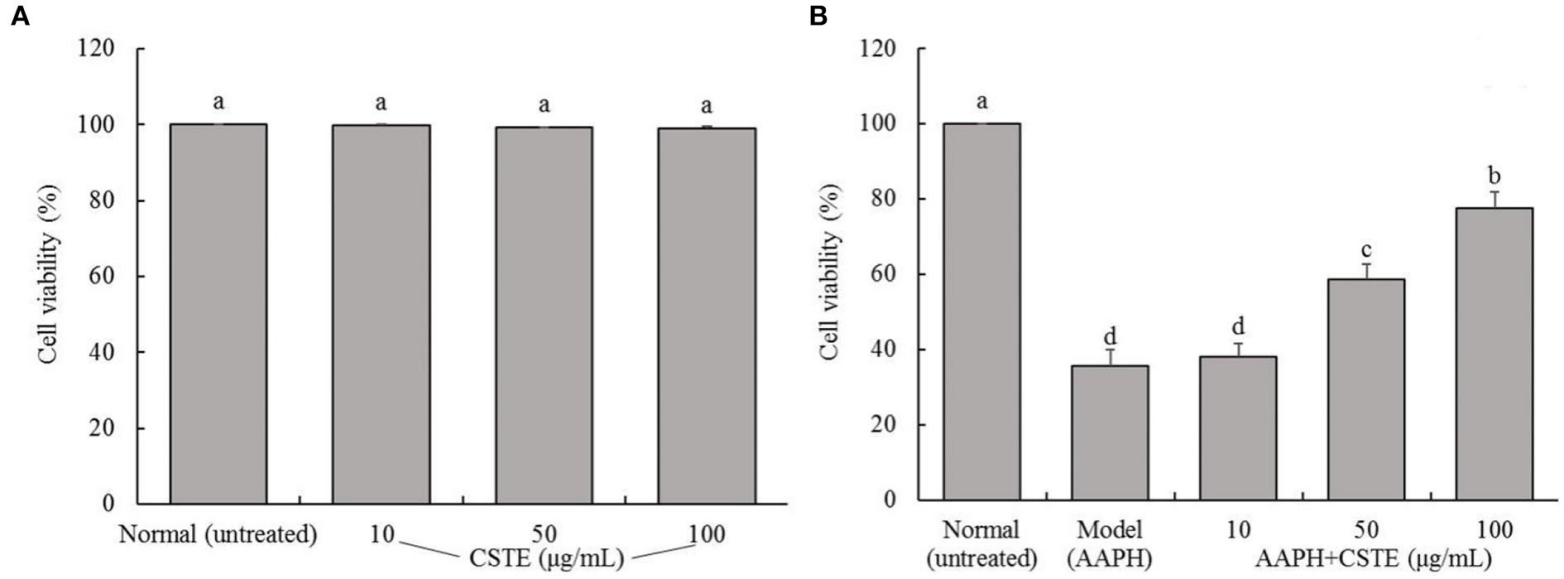
Survival rate of HL-1 cardiomyocytes, **(A)** cells not treated with AAPH, **(B)** AAPH induced oxidative damage of cells; CSTE: *Clerodendranthus spicatus* (Thunb.) C. Y. Wu extract. ^a−*d*^ according to Duncan's multi range test, the average values of different letters in the same column have significant differences (*P* < 0.05, *n* = 6).

The survival rates of the HL-1 cells after the CSTE treatments (10, 50, and 100 μg/mL) were > 90%, suggesting that the CSTE had no cytotoxic effect on the cells (Figure 2A). Additionally, direct exposure to AAPH (1 mmol/L) for 4 h significantly decreased the survival rates of the treated HL-1 cells when compared with the untreated group (*P* < 0.05). However, the survival rates of damaged cells increased when compared with untreated cells after treatment with the different CSTE concentrations for 24 h. Additionally, the protective effect was significantly enhanced (*P* < 0.05) as the CSTE concentration was increased to the 50 and 100 μg/mL concentrations (Figure 2B).

### Effects of the CSTE on MDA content and ROS levels in damaged HL-1 cells

**Figure 3:**
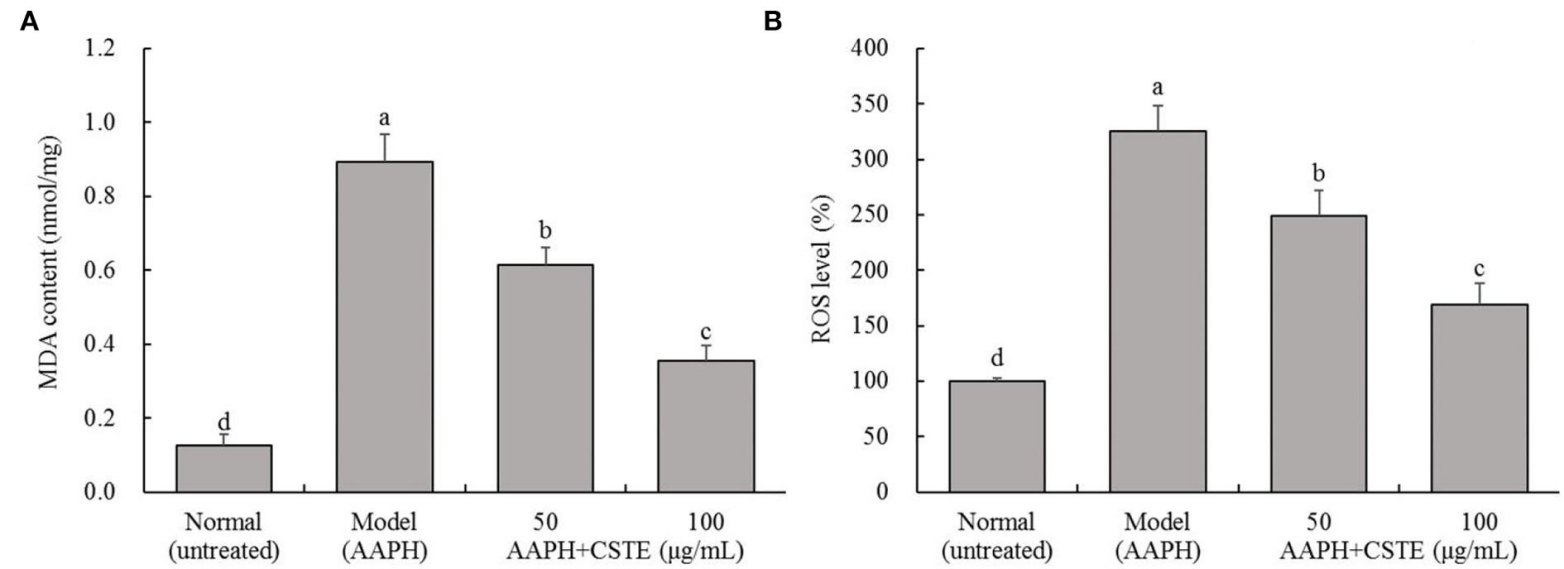
MDA content and ROS level of AAPH inducing oxidative damaged HL-1 cardiomyocytes, **(A)** MDA content, **(B)** ROS level; CSTE: *Clerodendranthus spicatus* (Thunb.) C. Y. Wu extract. ^a−*d*^ according to Duncan's multi range test, the average values of different letters in the same column have significant differences (*P* < 0.05, *n* = 6).

The MDA content in the HL-1 cells increased significantly after the 4-h AAPH treatment (1 mmol/L) compared with untreated cells (*P* < 0.05; Figure 3). However, MDA content decreased significantly after the CSTE treatments (50 and 100 μg/mL) (*P* < 0.05). The MDA content was lowest in the 100 μg/mL CSTE treatment. Additionally, the AAPH treatment significantly increased ROS levels in HL-1 cells (*P* < 0.05); however, the ROS levels in the damaged cells tended to decrease after the 24-h CSTE treatment (50 and 100 μg/mL). ROS activity was strongly inhibited when the CSTE concertation was 100 μg/mL.

### Effects of the CSTE on CAT, SOD, and GSH-Px activities in damaged HL-1 cells

**Figure 4:**
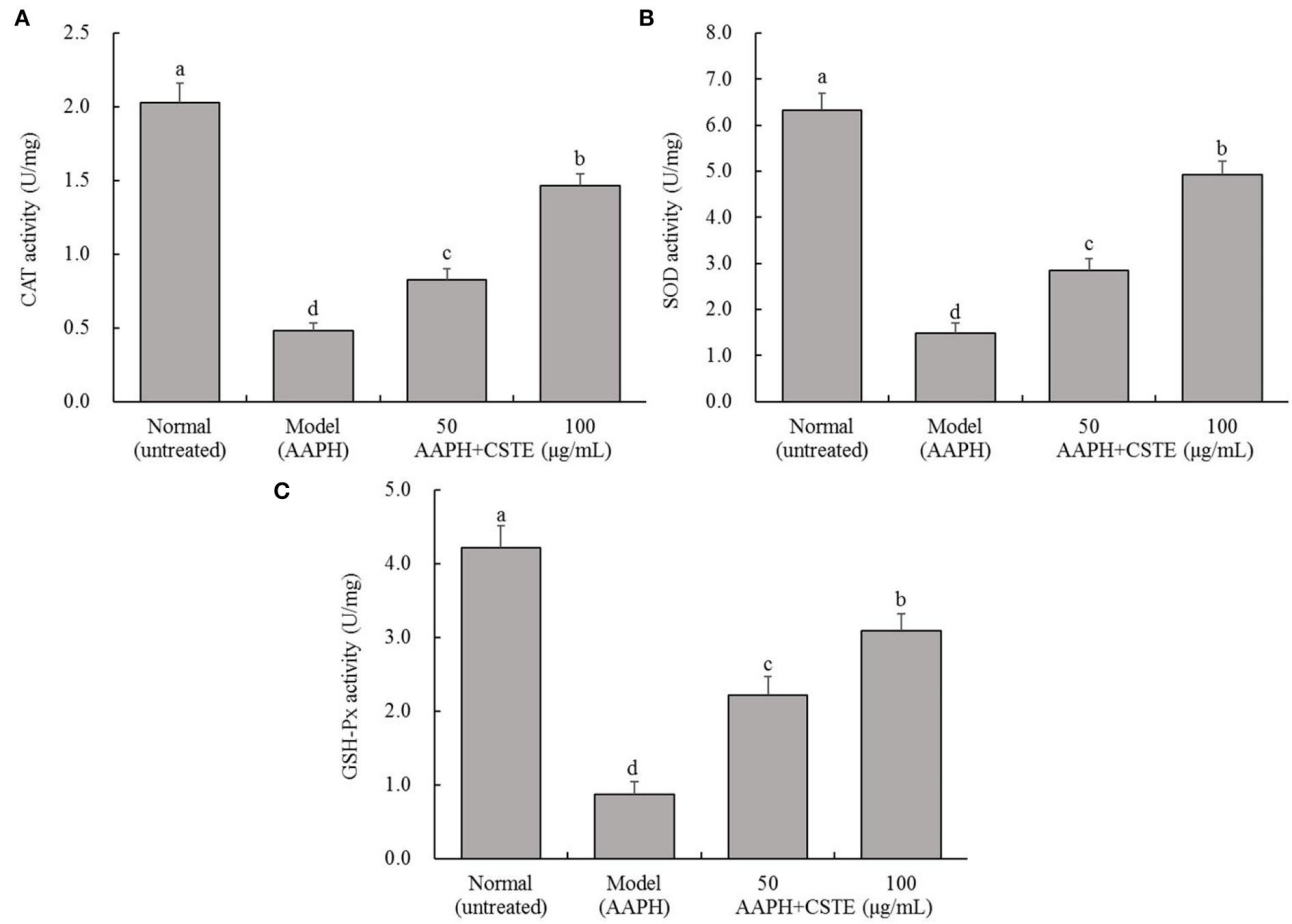
CAT, SOD, and GSH-Px activities of AAPH inducing oxidative damaged HL-1 cardiomyocytes, **(A)** CAT activity, **(B)** SOD activity, **(C)** GSH-Px activity; CSTE: *Clerodendranthus spicatus* (Thunb.) C. Y. Wu extract. ^a−*d*^ according to Duncan's multi range test, the average values of different letters in the same column have significant differences (*P* < 0.05, *n* = 6).

AAPH reduced CAT, SOD, and GSH-Px activities in the HL-1 cells (Figure 4). In contrast, antioxidant enzyme activities increased gradually in the damaged cells after the 24 h treatment with different CSTE concentrations (50 and 100 μg/mL), and a significant difference was observed when the damaged model groups were compared with untreated cells (*P* < 0.05).

### Effects of the CSTE on γ-GCS activity and GSH content in damaged HL-1 cells

**Figure 5:**
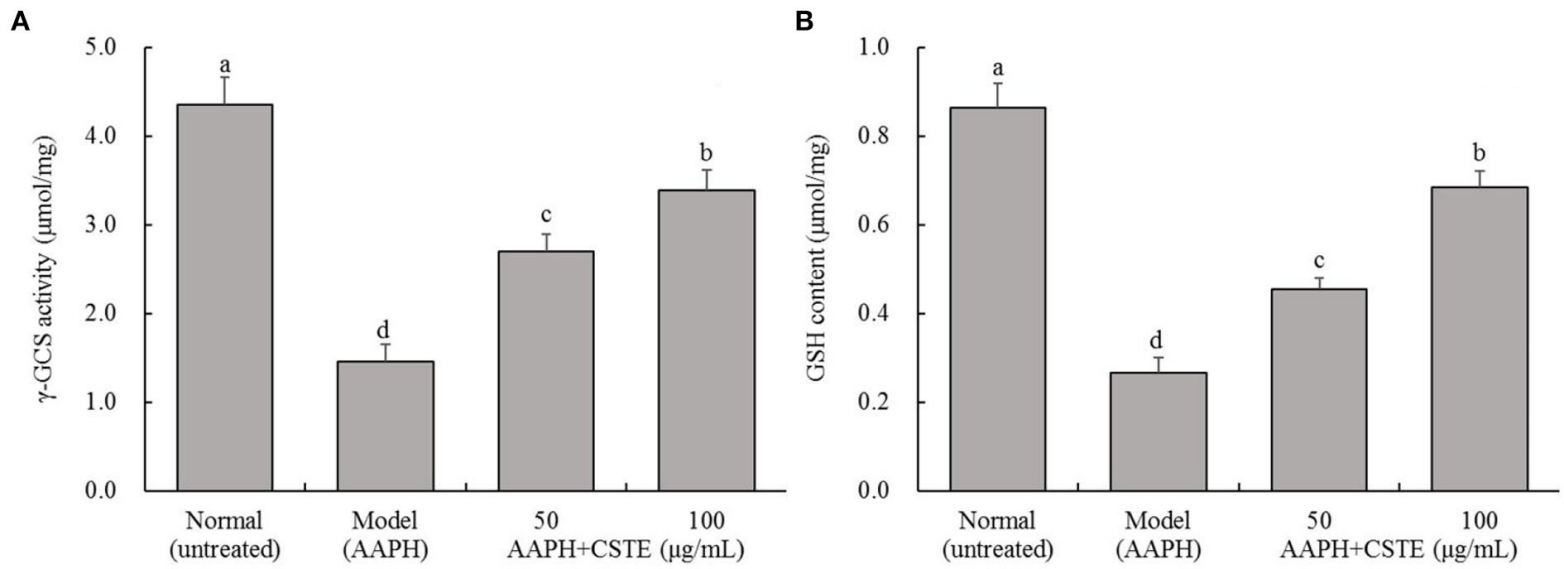
γ-GCS activity and GSH content of AAPH inducing oxidative damaged HL-1 cardiomyocytes, **(A)** γ-GCS activity, **(B)** GSH content; CSTE: *Clerodendranthus spicatus* (Thunb.) C. Y. Wu extract. ^a−*d*^ according to Duncan's multi range test, the average values of different letters in the same column have significant differences (*P* < 0.05, *n* = 6).

The AAPH treatment significantly inhibited intracellular γ-GCS activity and significantly reduced intracellular GSH content (*P* < 0.05; Figure 5). The γ-GCS content in damaged HL-1 cells increased gradually after the 24-h CSTE (50 and 100 μg/mL) treatments. Additionally, intracellular GSH content recovered and tended to increase. The higher CSTE dose (100 μg/mL) exerted significant effects on γ-GCS activity and GSH content in damaged HL-1 cells compared with the model group (*P* < 0.05).

### Effects of the CSTE on cell death in the oxidatively injured HL-1 cells

**Figure 6:**
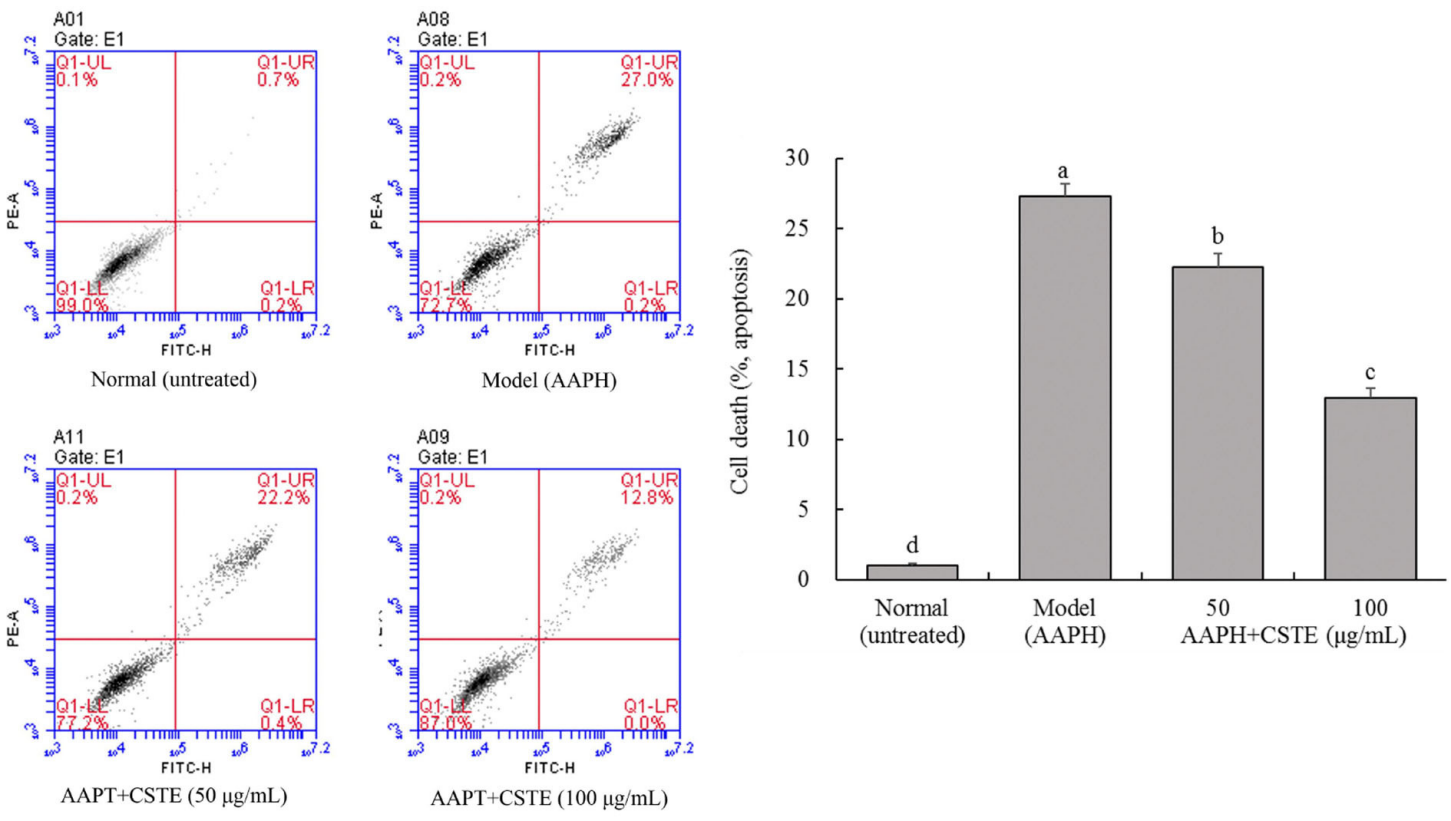
Flow cytometry of apoptosis HL-1 cells, CSTE: *Clerodendranthus spicatus* (Thunb.) C. Y. Wu extract. ^a−*d*^ according to Duncan's multi range test, the average values of different letters in the same column have significant differences (*P* < 0.05, *n* = 6).

Cell apoptosis is detectable by flow cytometry. The oxidatively damaged HL-1 cardiomyocytes appeared to have undergone apoptosis. About 27.3% of the cells in the model group underwent apoptosis, and these cells were considered dead. The CSTE significantly reduced apoptosis due to oxidative stress, thereby reducing the number of dead cells. The number of dead cells decreased with an increase in the CSTE concentration (Figure 6). Therefore, the CSTE significantly inhibited apoptosis in cardiomyocytes following oxidative damage, thereby reducing cell death.

### Effects of the CSTE on SOD1, GSH-Px, CAT, and Nrf2 gene expression levels in HL-1 cells

**Figure 7:**
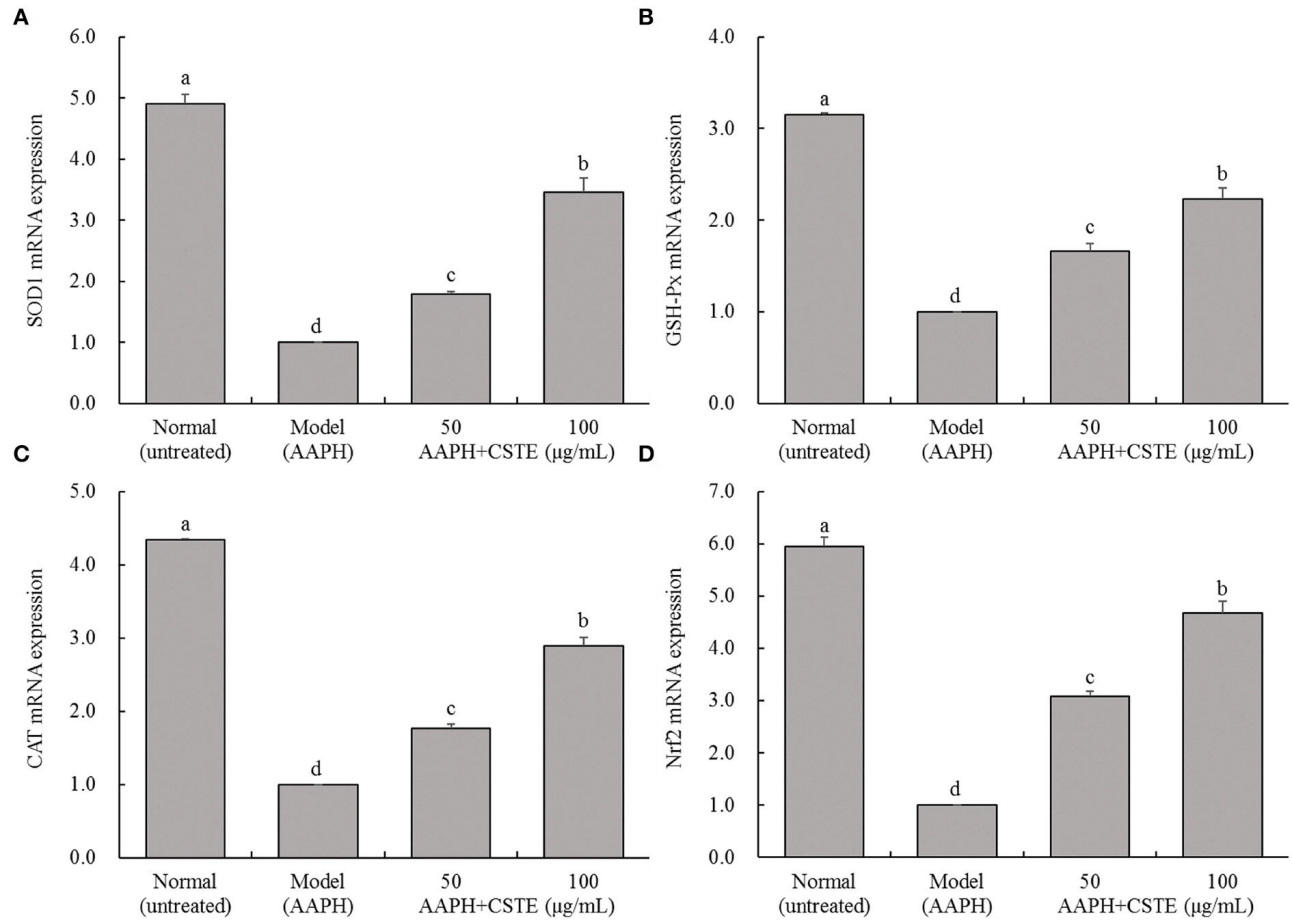
SOD1, GSH-Px, CAT and Nrf2 mRNA expression of AAPH inducing oxidative damaged HL-1 cardiomyocytes, **(A)** SOD expression, **(B)** GSH-Px expression, **(C)** CAT expression, (D) Nrf2 expression; CSTE: *Clerodendranthus spicatus* (Thunb.) C. Y. Wu extract. ^a−*d*^ according to Duncan's multi range test, the average values of different letters in the same column have significant differences (*P* < 0.05, *n* = 6).

The RT-fluorescent qPCR results showed that 1 mmol/L AAPH significantly decreased SOD1, GSH-Px, CAT, and Nrf2 mRNA levels in HL-1 cells (*P* < 0.05; Figure 7). However, these levels gradually increased after the CSTE treatment. The higher CSTE doses (50 and 100 μg/mL) exerted significant effects on SOD1, GSH-Px, CAT, and Nrf2 mRNA expression levels in injured cells compared with the model group (*P* < 0.05).

### Analysis of the active compounds in the CSTE

**Figure 8:**
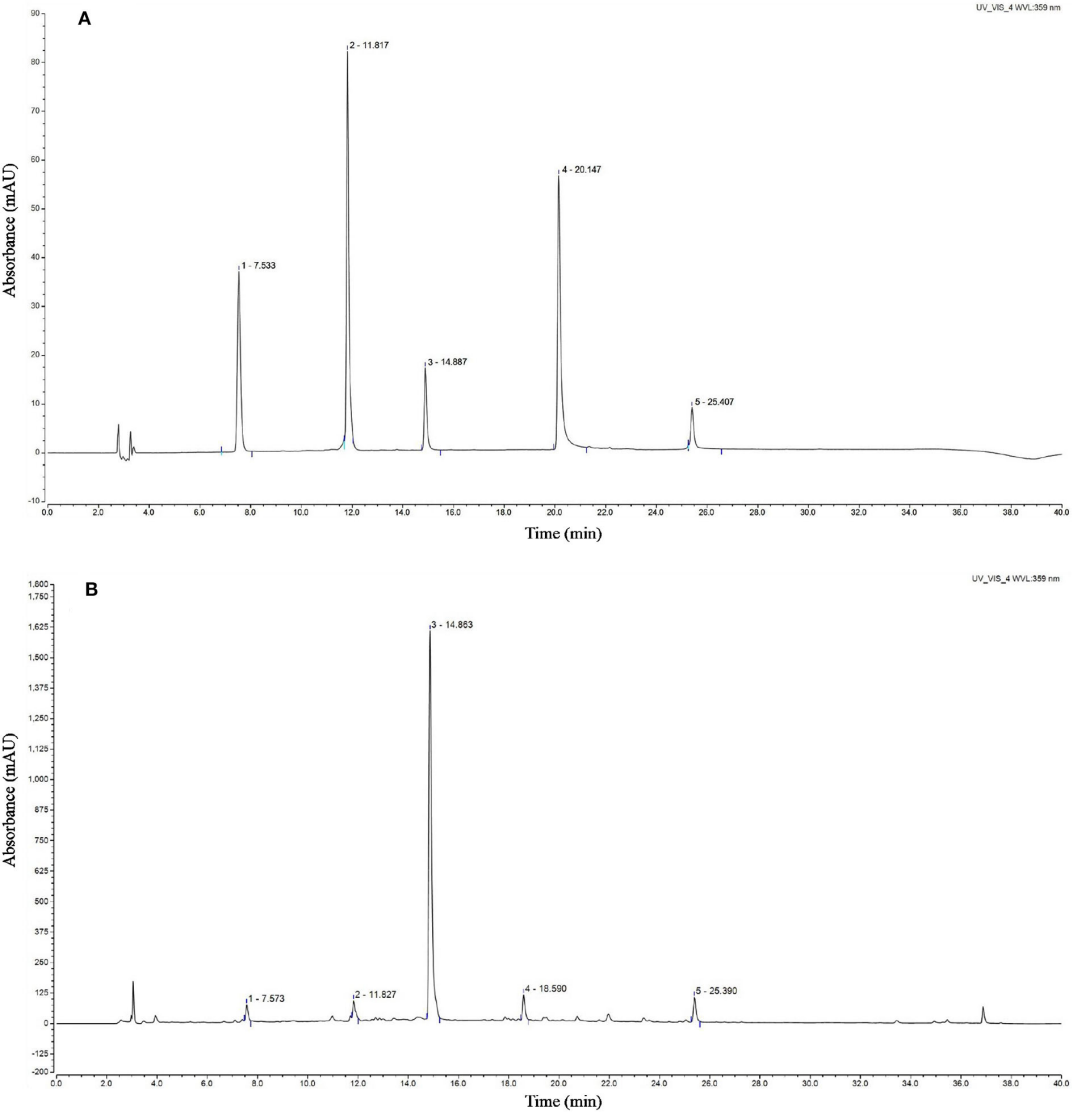
HPLC chromatogram of *Clerodendranthus spicatus* (Thunb.) C. Y. Wu extract, **(A)** standard chromatogram, **(B)** *Clerodendranthus spicatus* (Thunb.) C. Y. Wu chromatogram. 1: caffeic acid, 2: isoquercetin, 3: rosmarinic acid, 4; luteolin, 5: baicalin.

The chromatogram of the standards showed that the peak times of caffeic acid, isoquercetin, rosmarinic acid, luteolin, and baicalin were 7.533, 11.817, 14.887, 20.147, and 25.407 min, respectively. The HPLC analyses revealed that the CSTE contained caffeic acid, isoquercetin, rosmarinic acid, luteolin, and baicalin at the peak times of 7.573, 11.827, 14.863, 18.590, and 25.390 min, among which rosmarinic acid was the most important active compound (Figure 8).

## Discussion

Cardiomyopathy is a group of disorders that progressively impairs cardiac function due to structural changes in the lower chambers of the heart (i.e., ventricles) and impaired myocardial wall function. Cardiomyopathy is characterized by the aggravation of heart failure and serious arrhythmias, such as chest tightness, shortness of breath, dyspnea, wheezing, coughing foam-like sputum, enlargement of the liver and spleen, abdominal water, and edema of the lower limbs. Cardiomyopathy is a common disease in older people (16). Because older people frequently develop cardiomyopathy, the physiological changes in older people are closely related to the pathogenesis of cardiomyopathy. An imbalance of oxidative stress may be an important factor in the pathogenesis of cardiomyopathy. Excessive ROS accumulation is an important pathogenic factor in several chronic diseases. As important free radical donors, azo compounds, such as AAPH, spontaneously generate peroxy-free radicals, which damage DNA, proteins, lipids, and other biological macromolecules, leading to cell death. In this study, the survival rate of the HL-1 cells decreased significantly after exposure to AAPH (1 mmol/L). The survival rate was significantly higher than that of damaged cells treated with the CSTE for 24 h (*P* < 0.05). Additionally, excessive ROS generation induces lipid peroxidation of unsaturated fatty acids in cell membranes, thereby increasing MDA and 4-hydroxynonenal, which are toxic to cells (17, 18). Therefore, MDA is used as a marker of cell damage caused by oxidative stress (19). Treating the damaged HL-1 cells with the CSTE reduced total ROS levels and MDA content. The appropriate administration of antioxidants reduces ROS-induced lipid peroxidation in cardiomyocytes and prevents oxidative stress damage (20). A clinical study reported that eating an antioxidant-rich diet reduces oxidative stress levels in patients with chronic heart disease and eases the progression to end-stage heart disease (21). Furthermore, antioxidant therapy ameliorates the tissue damage caused by excessive oxidative stress in the heart tissue of patients with chronic heart disease (22). The water-soluble azo compound AAPH generates free radicals by thermal decomposition at physiological temperatures, which attack proteins or lipids to initiate peroxidation, ultimately leading to oxidative damage. Cell membranes are rich in unsaturated fatty acids, which are very sensitive to free radical-induced peroxidation, and can cause cell hemolysis (23). As the rate at which free radicals are generated by AAPH can be easily controlled and measured, the use of AAPH to induce hemolysis provides a good way to study free radical-induced membrane damage and detect myocardial cell damage.

The endogenous antioxidant enzymes (CAT, SOD, GSH-Px, and GST) and non-enzymatic GSH effectively fight against oxidative stress injury under normal physiological conditions. For example, SOD converts excess superoxide anions to H_2_O_2_, which is converted to water by CAT and GSH-Px (24). Additionally, GSH-Px uses GSH as a substrate to reduce H_2_O_2_ and alkane hydroperoxide levels and also reduces organic hydroperoxides (ROOH) to hydroxyl compounds (ROH) (25). GST helps GSH-Px remove excessive ROOH from the body. In our study, the activity of the antioxidant enzymes CAT, SOD, GSH-Px, and GST as well as γ-GCS content increased in damaged cells after pretreatment with different CSTE concentrations, GSH increased as well. Enhanced CAT and SOD activities prevented the damage caused by oxidative stress, inhibited lipid peroxidation reactions in cell membranes, and further alleviated oxidative stress damage to the cells (26, 27). As a major non-enzymatic antioxidant, GSH directly reduces toxic lipid peroxides and GSH-Px through H_2_O_2_ and indirectly inhibits free radical chain reactions, avoiding the cell damage caused by free radicals (28). γ-GCS is the rate-limiting enzyme in GSH biosynthesis and promotes GSH synthesis. After the ROS level increases, the activity of antioxidant enzymes and the expression of related genes in cells increases during the early stage of oxidative stress to resist oxidative damage (25, 26). Studies have shown that Nrf2 is the basis for defense against ROS. Nrf2 reduces the ROS level in the body and regulates the antioxidant enzymes to play a role in balancing oxidative stress. The CSTE also upregulated mRNA transcription levels of the major antioxidant enzymes in damaged HL-1 cells. By enhancing CAT, GSH-Px, and Nrf2 transcription levels, the CSTE improved the activity of intracellular total copper/zinc superoxide dismutase and alleviated oxidative stress injury in cells (29, 30).

Caffeic acid, isoquercetin, rosmarinic acid, luteolin, and baicalin have good antioxidant activities (31–35). Studies have reported that caffeic acid inhibits injury of adriamycin-induced cardiomyocytes (31). Roeosinic acid protects myocardial cells from hypoxia and reoxygenation injury, reduces cardiac fibrosis induced by stress, and inhibits myocardial hypertrophy induced by high glucose levels (36). An *in vitro* study showed that luteolin reduces the oxidative damage in cells caused by H_2_O_2_ (34). Luteolin significantly inhibits damage to H9c2 cardiomyocytes caused by an N_2_ saturated hypoxia chamber and Na_2_S_2_O_4_ hypoxic environment, and effectively maintains the balance between antioxidant defense and free radicals (37). Baicalein also protects the heart by inhibiting myocardial damage and infarction (38). Other studies have also shown that caffeic acid and rosmarinic acid components are the main components of *Clerodendranthus spicatus* (Thunb.) C. Y. Wu, but other studies have also shown that *Clerodendranthus spicatus* (Thunb.) C. Y. Wu also contains other components, such as methyl caffeate, ethyl caffeate, and vanillic acid, etc. (3, 39). Therefore, it can be seen that the composition of *Clerodendranthus spicatus* (Thunb.) C. Y. Wu from different origins is quite different, but the *Clerodendranthus spicatus* (Thunb.) C. Y. Wu used in this study are the only origins that can be used as medicines, and are representative.

## Conclusions

The combined actions of these five compounds may constitute the protective effects of the CSTE on the myocardium. There are very few studies on the physiological activity of CSTE, especially the research on the mechanism between the active ingredient and the physiological effect. In this study, the association and mechanistic effects of the physiological effects of the CSTE and the active ingredients were investigated. Our study provides a theoretical basis for the CSTE as a health food or traditional Chinese medicine with potent antioxidant capacity. However, as our study only involved *in vitro* analyses of HL-1 cells, specific *in vivo* CSTE-mediated molecular actions must be elucidated if the factors and antioxidant protective mechanisms in the cells are to be identified.

## Data availability statement

The original contributions presented in the study are included in the article/supplementary material, further inquiries can be directed to the corresponding author/s.

## Ethics statement

The animal study was reviewed and approved by the Ethical Committee of Chongqing Hospital of Traditional Chinese Medicine.

## Author contributions

YL wrote the first draft of the manuscript. JW collected the data and performed experiments. JJ and XL performed the experiments. MW contributed to conception and design of the study. All authors contributed to manuscript revision, read, and approved the submitted version.

## Funding

This research was funded by Natural Science Foundation of Chongqing, China (cstc2019jcyj-msxmX0662) and Senior Medical Talents Program of Chongqing for Young and middle-aged.

## Conflict of interest

The authors declare that the research was conducted in the absence of any commercial or financial relationships that could be construed as a potential conflict of interest.

## Publisher's note

All claims expressed in this article are solely those of the authors and do not necessarily represent those of their affiliated organizations, or those of the publisher, the editors and the reviewers. Any product that may be evaluated in this article, or claim that may be made by its manufacturer, is not guaranteed or endorsed by the publisher.
